# Vagus nerve stimulation as adjunctive therapy in patients with difficult-to-treat depression (RESTORE-LIFE): study protocol design and rationale of a real-world post-market study

**DOI:** 10.1186/s12888-020-02869-6

**Published:** 2020-09-29

**Authors:** Allan H. Young, Mario F. Juruena, Renske De Zwaef, Koen Demyttenaere

**Affiliations:** 1 Department of Psychological Medicine, Institute of Psychiatry, Psychology and Neuroscience, King’s College London & South London and Maudsley NHS Foundation Trust, Bethlem Royal Hospital, Beckenham, Kent UK; 2 LivaNova Belgium S. A, Zaventem, Belgium; 3 University Psychiatric Centre, KU Leuven, Leuven, Belgium

**Keywords:** Bipolar disorder, Difficult-to-treat depression, Major depressive disorder, Real-world setting, RESTORE-LIFE, Study design, Treatment-resistant depression, Vagus nerve stimulation therapy, VNS

## Abstract

**Background:**

Depressive illness is associated with significant adverse consequences for patients and their families, and for society. Clinical challenges are encountered in the management of patients suffering from depression whether they are designated difficult-to-treat or treatment-resistant. Prospective serial depression treatment trials have shown that less than 40% of patients with major depressive disorder remit with an initial pharmacotherapy trial, and a progressively smaller proportion of patients remit with each subsequent trial. For patients who suffer from difficult-to-treat depression (DTD), treatments should focus on patient-centred symptom control, patient functioning, and improving patient quality of life. Among the treatment options for patients with DTD is Vagus Nerve Stimulation (VNS) Therapy. VNS Therapy involves intermittent electrical stimulation of the left cervical vagus nerve and has been shown to be efficacious for long-term management of patients with DTD.

**Methods:**

RESTORE-LIFE is a prospective, observational, multi-site, global post-market study intended to assess short-, mid-, and long-term effectiveness and efficiency outcomes in a ‘real-world’ setting among patients with DTD treated with adjunctive VNS Therapy. A minimum of 500 patients will be implanted with a VNS Therapy System at up to 80 global sites. Eligible patients will participate in a baseline visit between 1 and 6 weeks before device implant and will be followed for a minimum of 36 months and a maximum of 60 months. The diagnosis of depression and comorbid disorders will be determined using the Mini-International Neuropsychiatric Interview (MINI). The primary endpoint is response rate, defined as a decrease of ≥50% in Montgomery Åsberg Depression Rating Scale (MADRS) total score from baseline to 12 months post-implant.

**Discussion:**

A standardized approach in the management of DTD may not be appropriate for the treatment of such a complex heterogenous patient population. This study has been designed to evaluate whether VNS Therapy meaningfully improves and sustains clinical and depressive symptom outcomes in patients with DTD. This study will investigate the durability of VNS response in DTD and utility of VNS for long-term disease management of DTD. In addition, the study results will potentially clarify clinical, functional, and health economic questions in a real-world patient population with DTD.

**Trial registration:**

ClinicalTrials.gov NCT03320304. Registered 25 October 2017

## Background

Depressive illness (‘depression’) is one of the major healthcare issues in the twenty-first century and is ranked by the World Health Organization as the single largest contributor of years lived with disability worldwide [1]. Depression is associated with a high burden of disease and has significant adverse consequences for patients, their families, and for society due to impaired daily functioning, impaired social relationships, and financial burdens [2, 3]. Depression is also associated with a significant increased mortality risk due to comorbid medical disorders and increased suicidality [4, 5].

Currently, there are three common treatment modalities with substantial evidence of effectiveness in the treatment of major depressive disorder [6, 7]. Pharmacotherapy with antidepressant drugs is usually the first-line treatment for major depression and prospective serial depression treatment trials have demonstrated that about 40% of patients will not achieve and sustain remission with an initial pharmacotherapy trial, and a progressively smaller proportion of patients remit with each subsequent trial, until the remission rate after a fourth antidepressant trial is between 10 and 15% [8–10]. Manual-based psychological therapy is another common treatment modality for major depression, with the most persuasive evidence for cognitive behavioural therapy [11]. Several neurostimulation strategies ─ principally electroconvulsive therapy (ECT) ─ are also available for individuals who are insufficiently responsive to pharmacotherapy and/or psychosocial interventions, with clinicians reserving ECT for patients with psychotic depression or when an immediate response to treatment is warranted [12].

Patients who do not adequately respond to multiple therapeutic interventions are considered to have difficult-to-treat depression and constitute about 15 to 20% of patients with major depressive disorder [13–16]. Such patients have significantly higher utilisation of health care resources, such as inpatient care, pharmaceutical drugs, and increased medical utilization for non-psychiatric conditions compared to patients with depression who successfully remit after initial attempts with standard treatment options [17]. When direct costs such as health care costs and indirect costs such as those resulting from lost productivity are combined, it has been estimated that depression is the second most costly medical condition in developed countries [18, 19].

Describing such a patient population as experiencing difficult-to-treat depression (DTD) is preferred over the previous term of treatment-resistant depression (TRD) as it has been suggested that TRD negatively categorizes a patient’s condition, whereas, the DTD term is a holistic and more clinically useful collaborative construct that recognizes the complexities of creating an effective treatment plan and may encourage patients and clinicians to consider a broader range of treatment options [20, 21].

Several new treatment modalities for DTD have emerged such as (es) ketamine, neurosteroids, and additional forms of neurostimulation based on electrical or magnetic impulses: Vagus Nerve Stimulation Therapy (VNS Therapy®) and repetitive transcranial magnetic stimulation (rTMS) [22].

VNS Therapy is an approved adjunctive treatment option for patients with DTD [23–25]. In 2001, VNS Therapy received CE marking in Europe for the treatment of chronic or recurrent depression in patients who are in a treatment-resistant or treatment-intolerant major depressive episode. VNS Therapy was approved in the United States in 2005 by the US Food and Drug Administration for the adjunctive long-term treatment of chronic or recurrent depression for patients 18 years of age or older who are experiencing a major depressive episode and have not had an adequate response to four or more adequate antidepressant treatments.

**Fig. 1:**
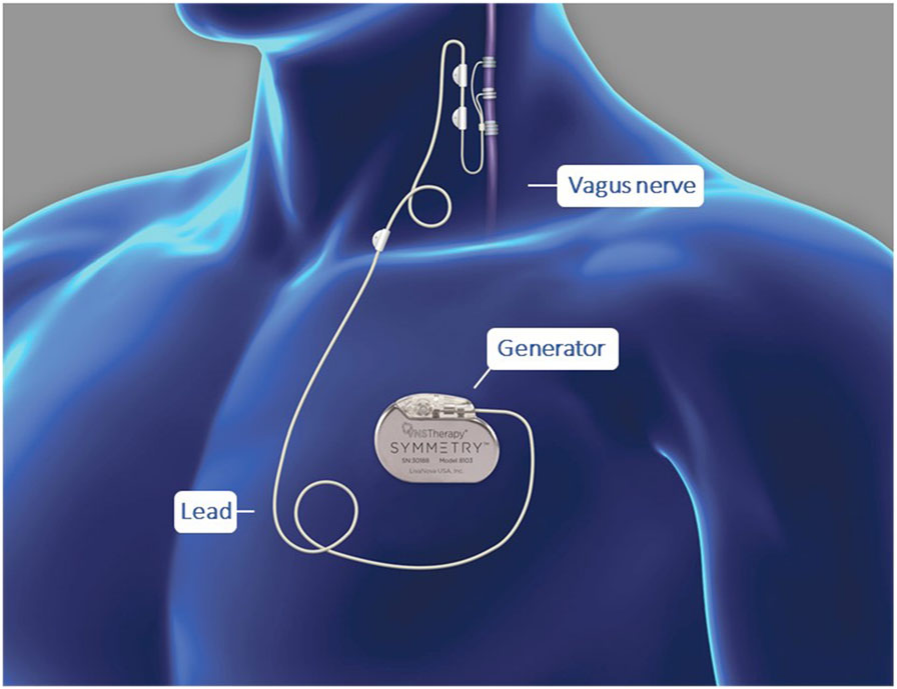
schematic representation of the VNS therapy system. Study participants will be implanted with the VNS Therapy system that includes a small pulse generator surgically implanted subcutaneously in the thoracic area (usually the left side) and a thin, flexible wire called a lead that connects to the vagus nerve (Fig 1). Mild intermittent electrical stimulation is delivered to the vagus nerve eliciting action potentials in the vagal fibres that synapse in the brainstem. Clinicians are able to program the system using an external programming device relying on attentive patient-specific titration to select the right combination of adjustable parameters (output current, frequency, pulse width, and signal ON and OFF times) to attain full activation of the vagus nerve while minimizing stimulation-induced side effects. Additional information is provided in the VNS Therapy Physician’s Manual and the Implant Manual

VNS Therapy consists of an implanted pulse generator connected to a helical electrode that wraps around a patient’s left cervical vagus nerve (Fig. 1). This system delivers mild intermittent electrical stimulation to the vagus nerve eliciting action potentials in the vagal fibres that synapse in the brainstem. The anode and cathode electrodes are positioned on the vagus nerve such that the anode is distal to the patient’s head forming an anodal block and ensuring stimulation of afferent fibres. The afferent sensory fibres of the vagus nerve terminate at the nucleus tractus solitarius which in turn projects to the locus coeruleus. The locus coeruleus contains norepinephrine-containing neurons with connections to various regions, including the thalamus, the hypothalamus, the orbito-frontal cortex, and the cerebellum.

The stimulation of the left vagus nerve has been shown to induce widespread bilateral effects in areas of the brain implicated in affective regulations that are also responsible for modulation of key neurotransmitters such as serotonin and norepinephrine.

Several large clinical studies have evaluated VNS Therapy in patients with depression since the market approval and have shown that the treatment is efficacious for long-term patient management; and a meta-analysis demonstrated that VNS has an acceptable adverse event profile [25–28]. These studies also demonstrated that the assessment of depression outcomes has to be longitudinal and of sufficient duration an improved clinical response is observed within 6 to 24 months following implantation with the mean time-to-first response of 12 months (defined as a 50% reduction in baseline depression assessment scores). Additional studies of VNS Therapy in the treatment of depression suggest that: (1) VNS Therapy has a positive effect on overall mortality and suicidality in DTD [29]; (2) VNS Therapy has clinically meaningful improvements in quality of life in patients with unipolar as well as bipolar disorder, even when the response is below the classic 50% reduction from baseline score [30]; (3) VNS Therapy improves cognitive and clinical measures in DTD [31]; (4) VNS Therapy is associated with localized changes in the brain as evidenced by functional brain imaging techniques [32]; and (5) patients who fail ECT may respond to VNS [33].

Despite these clinical evidence of benefits in the treatment of depression, VNS Therapy remains to be used as a standard add-on treatment to antidepressants, mood-stabilizing agents, or other augmentation agents in persons with DTD. Several reports have found either an earlier or more frequent benefit (in both response and remission rates) when VNS Therapy was added to treatment as usual (TAU) as compared to TAU alone [25–27].

Currently there is no consensus on the required number of medication failures prior to consideration of VNS Therapy as a treatment option in patients with DTD; and guidelines are defined by each country or reimbursement agency. A recent international consensus publication from McAllister-Williams et al. has suggested identification and management of DTD based on consideration of DTD as a chronic condition and proposed defining DTD as ‘depression that continues to cause significant burden despite usual treatment efforts’ [20]. The authors also described a practical framework that looks beyond clinical symptom control to assess treatment success in patients with DTD and focuses on patient-centred expectations of meaningful improvements in symptom control, cognition, daily psychosocial functioning, and overall quality of life. Furthermore, the authors stated that DTD may be suspected or diagnosed in some patients following one or two treatment failures, especially if comorbidities and concomitant medications are barriers to prescribing additional treatment options.

### Study aims

The aim of the RESTORE-LIFE study (a global pRospective, multi-cEnter, obServational post-markeT study tO assess shoRt-, mid-, and long-term Effectiveness and efficiency of VNS Therapy® as adjunctive therapy in reaL-world patIents with diFficult-to-treat dEpression) is to assess whether adjunctive VNS Therapy meaningfully improves and sustains (short-, mid-, and long-term) clinical outcomes of patients with DTD in a ‘real-world’ (standard of care) patient population from various countries. The study will also investigate the durability of response to VNS Therapy in DTD and overall long-term disease management of these patients based on the changes in mood, severity of depression, mania, quality of life, psychosocial impairment, cognition, anxiety, suicidality, adjunctive antidepressant treatment usage, and safety assessments. Exploratory aims include assessment of positive affect, negative affect, hedonic tone, cognitive functioning, overall functioning, meaningfulness of life, and happiness; and a health economics approach based on resource consumption analysis.

## Methods

### Study design

This prospective, observational, multi-site, global post-market study will collect data on a minimum of 500 patients implanted with a VNS Therapy device at up to 80 global study sites. The primary objective of the study is to assess short-, mid- and long-term clinical outcomes in patients with DTD treated with adjunctive VNS Therapy. The primary endpoint of response rate is defined as a decrease of ≥50% in Montgomery Åsberg Depression Rating Scale (MADRS) total score from baseline to 12 months post-implant, and the analysis will include patients who were experiencing a major depressive episode at the time of enrolment (i.e. a baseline MADRS total score ≥ 20).

The schedule of assessments is provided in Table 1. Each study participant will participate in a baseline visit between 1 to 6 weeks before the VNS Therapy implantation surgery. Once implanted with the VNS Therapy device, participants will be followed for a minimum of 36 months and a maximum of 60 months. Participants will report to the site for study follow-up assessments and the study visits will coincide with the standard medical care for patients treated with VNS Therapy. The study physician may schedule additional standard of care visits to manage the participant’s depression as necessary and according to the study physician’s standard practice.

**Table 1:** Schedule of assessments

Visits	Baseline	Implant (Day 0)	Titration visits (as needed following implant)	Follow-up visits (at 3, 6, 9, 12, 15, 18, 24, 30, and 36 months) ^**a**^]	Long-term follow-up (every 6 months until 60 months or study ends)
Allowed visit window	6 weeks to 1 week before implant	n/a	n/a	± 45 days	± 60 days
Informed consent	X				
Assessment of eligibility criteria	X				
VNS Therapy implant/revision/explant		X	As needed		
VNS Therapy parameter settings		X	X	X	X
Device deficiency monitoring (if applicable)		X	X	X	X
Study assessments					
MINI	X				
Modified ATHF	X				
Medical history, demographics, and baseline patient characteristics	X				
MADRS	X			X	X
QIDS-SR (not collected at 3, 9, and 15 months)	X			X	X
Q-LES-Q-SF (not collected at 3, 9, and 15 months)	X			X	X
EQ-5D-5L (not collected at 3, 9, and 15 months)	X			X	X
WPAI:D	X			X	X
LAPS	X			X	X
ASRM ^b^	X			X	X
Cognition test (THINC-it®) (not collected at 3, 9, and 15 months) ^b^	X			X	X
GAD-7 (not collected at 3, 9, and 15 months) ^b^	X			X	X
Health Care Utilization Form (not collected at 3, 9, and 15 months) ^c^	X			X	X
Antidepressant treatments ^d^	X			X	X
Adverse events monitoring	X	X	X	X	X
Study completion/termination record ^e^			At study exit		

^a^ QIDS-SR, Q-LES-Q-SF, EQ-5D-5L, THINC-it, GAD-7, and Health Care Utilization Form are not collected at 3, 9, and 15 months

^b^The ASRM, THINC-it, and GAD-7 are optional assessments to be completed at selected sites only and will be upto the investigators’ clinical judgment to decide which participants complete the assessments

^c^Health care utilization data (eg, number of depression-related emergency room visits and/or hospitalizations) will be obtained where possible (patient reported and/or based on medical records, whichever is available). More detailed information may be derived from a national health care database, if available, and if the patient provides consent to collect this data

^d^ Antidepressant medications and psychotropics, ECT, rTMS, and/or psychotherapy (such as interpersonal therapy and cognitive behavioural therapy)

^e^ Including participant satisfaction record (if applicable)

All participants will exit the study after completing 60 months of post-implantation follow-up, or at study end (whichever comes first). The study may be stopped when the last participant has reached the 36 months of follow-up. Study end will be communicated to all participating sites. The study will be considered completed when all enrolled participants complete the study completion/termination record.

### Study sites and recruitment

Eligible patients will be recruited from patients who have been referred for treatment with VNS Therapy at sites that offer VNS Therapy for the treatment of DTD per standard of care. Up to 80 sites worldwide may participate in this study. Participating clinical study sites will be carefully selected and must be specialty psychiatry centres with experience treating patients with DTD (ie, have experience with VNS Therapy, ECT, or rTMS). Recruitment materials, such as posters, folders, advertisements, will only be used after obtaining the applicable approvals from ethics committees and/or competent authorities.

### Participants and eligibility

The study population will comprise of a global ‘real-world’ (standard of care) patient population with DTD referred for treatment with VNS Therapy; therefore, the inclusion criteria as presented below are minimal. The study physicians should reference the indications and contra-indications as specified in the VNS Therapy Physician’s Manual that is applicable in their region.

Eligible participants are adults of 18 years of age or older with a documented primary diagnosis of chronic (> 2 years) or recurrent (2 or more prior episodes) major depressive episode that has not adequately responded to an adequate number of antidepressant treatments, as per local medical standards.

The current depressive episode and comorbid disorders will be determined using the Mini-International Neuropsychiatric Interview (MINI) which is a physician-rated neuropsychiatric instrument that will be used to confirm the diagnosis of eligible mood disorders including major depressive disorder, bipolar I disorder, and bipolar II disorder, each in the depressed phase, and identify comorbid disorders [34–36]. In addition, the course of the mood disorder must evidence a high likelihood of the need for long-term treatment as evidenced by the current major depressive episode lasting at least two years or the history of at least two prior major depressive episodes.

The level of DTD must also be substantial and clear as evidenced by the failure to respond adequately to standard psychiatric management. These failed antidepressant trials will be verified using the modified Antidepressant Treatment History Form (ATHF) [37]. This is a clinician-rated scale used to collect information on the adequacy and response to previous antidepressant treatments, such as medications, trials of ECT, rTMS, psychotherapy, and newer antidepressants and medication combinations.

Additional key eligibility criteria include currently receiving at least one antidepressant treatment (such as antidepressant drug, maintenance ECT, or formal psychotherapy including supportive psychotherapy) or mood stabilizing treatment for bipolar patients (such as lithium, anticonvulsants, or atypical antipsychotics); and ability and willingness to comply with the frequency of (outpatient) clinic visits and to reliably complete all the evaluations as specified in the study protocol. Based on the nature of their disease, the following patients should be excluded from study participation: patients with mental retardation, current severe or significant substance/alcohol abuse, diagnosis of one or more schizophrenia-spectrum or other psychotic disorders, diagnosis of borderline or severe personality disorder as determined by clinical judgment which, in the investigator’s opinion, would significantly interfere with study participation.

### Intervention (VNS therapy implant procedure and programming)

Per standard of care, all participants will be implanted with a commercially available implantable VNS Therapy System (manufactured by LivaNova) with generator, lead, and external programming system. The VNS Therapy Physician’s Manual and the Implant Manual contain instructions regarding the implantation procedure and initial device parameters/diagnostics. Surgeons performing the implantation procedures should have the appropriate qualifications and training to perform the surgery. VNS Therapy consists of a pulse generator which is surgically implanted subcutaneously in the thoracic area (usually the left side) and delivers intermittent electrical signals to the vagus nerve via a lead that is partially wrapped around the vagus nerve in the mid-cervical region. The electrical signals are relayed to various regions of the brain. The system is programmed via an external programming device (see Fig. 1).

Following device implantation, the device must be switched ON and titrated to clinical efficacy as per standard medical care. Clinicians program the system using an external programming device and rely on attentive patient-specific titration to select the right combination of adjustable parameters (output current, frequency, pulse width, and signal ON and OFF times) to attain full activation of the vagus nerve while minimizing stimulation-induced side effects. Usually stimulation is delivered intermittently, typically 30 s ON, 5 min OFF. The rate of titration varies by patient and their response until a therapeutic dose has been established; all sites are encouraged to reach a stimulation of minimally 1 mA (considered minimal therapeutic dose) at a duty cycle of minimally 10%. This recommendation is based on previous findings that a higher delivered charge predicted greater sustained antidepressant effects [38].

The sponsor has developed detailed metrics on the titration procedure to assist clinicians reach the optimal therapeutic dose of VNS for individual patients. Patients who are under-dosed and do not achieve the therapeutic dose of VNS will be identified via a so-called ‘risk-based study monitoring’ method. The sponsor will contact sites that have patients below therapeutic dose and if needed the sponsor will provide technical support to the principal investigators on titrating VNS parameters to reach the minimal therapeutic dose as reasonably possible.

All data regarding the device implant and parameter settings will be recorded.

The sponsor/device manufacturer’s personnel will be available as needed to provide technical support to principal investigators and as requested, support may include investigative site training, on-site troubleshooting, providing clarifications to study sites concerning the operation of the VNS Therapy device (including programmers and other support equipment), demonstrating the assembly and operation of the VNS Therapy device, and clarify device operation or output.

Additional information is provided in the VNS Therapy Physician’s Manual and the Implant Manual.

### Additional quality control measures

Prior to investigational site activation or involvement in study activities, training will be provided to the site personnel who will be involved in study activities. The study physician or designees must conduct the study as described in the protocol except for an emergency in which proper care of the participant requires immediate action. Any deviation from the protocol must be reported promptly.

The study physician or designee is responsible for maintaining adequate and accurate medical records from which all data required per protocol is captured into the electronic case report forms. The patient-reported outcome questionnaires can be completed by the participant directly on a tablet computer using an electronic patient reported outcome (ePRO) system.

Monitoring of the clinical study will be an interactive process to ensure that high-quality data is obtained and that the study is conducted in compliance with the protocol, applicable laws, regulations, and good clinical practice guidelines. A risk-based monitoring approach will be followed to provide flexibility for on-site monitoring visits and centralized monitoring methods.

### Effectiveness and efficiency outcome measures and assessments

The RESTORE-LIFE study will conduct a series of assessments designed to measure the response rate, durability of response to VNS Therapy in DTD, and overall long-term disease management of these patients based on changes in mood, severity of depression, mania, quality of life, psychosocial impairment, cognition, anxiety, suicidality, adjunctive antidepressant treatment usage, and safety assessments. The assessments will be conducted at regular time intervals during standard medical care as specified in the Schedule of Assessments (Table 1) to evaluate changes in clinical symptoms over time in response to adjunctive VNS Therapy. The primary, secondary, and exploratory outcome measures along with information on the related scales and questionnaires are listed in Table 2.

**Table 2:** List of effectiveness and efficiency outcome measures

Outcome measures	Measurement details with clinician-rated and patient-reported scales
**Primary outcome measure**	
Rate of response	Decrease of ≥50% in MADRS total score from baseline to 12 months post-implant. The MADRS is a physician-rated instrument to assess the severity of depressive symptoms during the past week [39, 40].
**Secondary outcome measures**	
Duration of response	The difference between the first recorded date of post-baseline MADRS score when response is achieved (based on a decrease in baseline MADRS score of ≥50%) and the first date at which the MADRS score reaches a level of < 40% from baseline.
Duration of response	Change in MADRS score over time
Duration of response	Cumulative percentage of first-time responders (based on reduction in baseline MADRS score of ≥50%) and cumulative remission (based on MADRS score of ≤9 at any post-baseline visit) over time.
Changes in mood, depression, and mania scores	This will be based on changes in MADRS, QIDS-SR, and ASRM. The QIDS-SR questionnaire measures symptoms of mood and depression [41]. The ASRM measures the presence and severity of manic and hypomanic symptoms, specifically in patients diagnosed with bipolar disorder [42]. Note: ASRM is an optional assessment.
Changes in quality of life and psychosocial impairment	The WPAI:D questionnaire measures impairments in work and activities in depression [43]. The Q-LES-Q-SF scale measures the degree of enjoyment and satisfaction experienced during the past week [44]. The EQ-5D-5L questionnaire measures generic health status and quality of life [45].
Changes in suicidality	Based on Item Number 10 of MADRS and Item Number 12 of QIDS-SR.
Changes in antidepressant treatments	This will include data on drug dosage and type of antidepressant medications, and duration and intervals of maintenance ECT, rTMS, and/or psychotherapy.
Changes in cognition	THINC-it® Tool includes the 5-item PDQ-5, in addition to 4 traditional cognitive assessments which have been reconfigured for computer-based administration. Thus, it assesses both a patient’s subjective assessment as well as key objective measures of cognitive function [46]. Note: THINC-it is an optional assessment.
Changes in anxiety	The GAD-7 measures severity of generalized anxiety disorder [47]. Note: GAD-7 is an optional assessment.
**Exploratory outcome measures**	
Changes in positive affect, negative affect, hedonic tone, cognitive functioning, overall functioning, meaningfulness of life, and happiness with adjunctive VNS treatment	The LAPS questionnaire assesses negative affect, positive affect, hedonic tone, and meaningfulness of life [48].
Healthcare resource utilization analysis	Healthcare utilization data will be used to assess the main sources of resource utilization associated with DTD management per patient who have been subsequently treated with VNS Therapy. The collected data will include the number of depression-related emergency room visits leading to hospitalizations, antidepressant medication usage, and adjunctive antidepressant treatments before and after the start of VNS Therapy.

The patient-completed self-report assessments will be completed by participants who are able to complete the assessments with minimal assistance

The ASRM, THINC-it Tool, and GAD-7 are optional assessments that will be completed at selected and interested sites only, and participants will be selected to complete the assessments based on the study physician’s clinical judgment

Note that only validated scales and questionnaires are being used in this study. For each participating country, the sponsor obtains fully licensed and validated translations of the scales and questionnaires in the local languages.

Some of these scales and questionnaires will be completed by the study participants, and the other scales will be completed and assessed by the study investigator/physician or qualified mental health care professional with the training and clinical experience necessary to conduct these assessments. Care will be taken to ensure that the assessments are conducted in a similar manner and condition at all visits for a given participant.

### Healthcare resource utilization

An exploratory analysis will be conducted with the aim of identifying the main sources of resource utilization related to patients with DTD who are subsequently treated with VNS Therapy (Table 2). This exploratory analysis is not being planned to demonstrate any resource consumption savings associated with VNS therapy as the study is not designed for this purpose.

To enable a calculation of the expenditures associated with the use of the VNS Therapy system, the following data will be collected (prior to enrolment and during study): number of emergency room visits (depression related or not); number of depression related emergency room visits leading to hospitalization; number, dosage, and type of antidepressant medications and psychotropics; number of adjunctive antidepressant treatments such as ECT, rTMS, and/or psychotherapy (such as interpersonal therapy and cognitive behavioural therapy). The data will be obtained from patient’s self-reports and/or medical records, as available.

For those patients who provide consent to collect additional data and who are enrolled in a country with a national healthcare database, a more detailed resources consumption analysis will be conducted (such as number of depression related general practitioner, emergency room visits and/or outpatient visits).

### Safety assessments

All participants will be monitored for safety and evaluated for any depressive symptoms (including suicidal ideation) by completing study assessments throughout the study. Additionally, the participants will be instructed to contact their physician in case of worsening depression/suicidality. The sponsor is responsible for the ongoing safety evaluation of the VNS Therapy system, review of reported adverse events, investigation of unanticipated serious adverse device effects, and notification of regulatory authorities per applicable requirements.

For safety evaluation, data will be collected on adverse events, serious adverse events, VNS Therapy related adverse events and serious adverse events (including the use of the device), unanticipated adverse device effects, and device deficiencies. Adverse events will be followed until (a) resolution, (b) stabilization, (c) the end of the study, or (d) if a study physician and/or sponsor determines that a device-related adverse event must be followed until its conclusion which extends beyond the end of the study.

The study physicians should reference the VNS Therapy Physician’s Manual for a list of anticipated adverse events that have been frequently reported in clinical studies. Any serious adverse device effect not listed in the Physician’s Manual will be considered as unanticipated.

### Concomitant treatments

During participation in the study, participants will not be required to stop or change any concomitant medications or treatments. All adjunctive treatments for the treatment of depression are allowed per physician’s clinical judgment based on site’s standard of care.

Participants enrolled in this study must be receiving at least one antidepressant treatment (ie, antidepressant drug, maintenance ECT, or formal psychotherapy including supportive psychotherapy) or mood-stabilizing treatment for bipolar patients (such as lithium, anticonvulsants, or atypical antipsychotics). Concomitant use of antidepressant medications and psychotropics, non-pharmacologic antidepressant treatments for depression, and inpatient hospitalizations will be recorded.

### Sample size and statistical analysis considerations

As this is an observational study designed to describe the characteristics of VNS Therapy in a real-world setting, the statistical analysis of the study outcomes will focus on a combination of descriptive and exploratory statistics, rather than confirmation of formal hypothesis testing; that is, the provision of two-sided 95% confidence intervals rather than *p*-values. As an additional consequence, the emphasis provided in the study will be on the precision of the estimate. A sample size of 500 patients is sufficient to provide higher precision of the estimates in term of width of the 95% confidence interval for the key study endpoints:

- For the primary endpoint based on MADRS, the width of the 95% CI will be of 7.8% assuming an observed response of approximately 25%.
- For the secondary endpoint based on Q-LES-Q-SF response (and the available Q-LES-Q-SF data obtained in the D-23 study), the width of the 95% CI will be 8.9% assuming an observed response of approximately 50% [49].

Enrolled analysis population will include all participants who signed the informed consent and who satisfy the study eligibility criteria. Safety analysis population will include all enrolled participants who were implanted with the VNS Therapy system and received at least one dose of stimulation. The full analysis set population will include all participants in the safety analysis population with evaluable baseline device data; that is, patients who have the device implanted and received at least one dose of stimulation. As such, for this study, the full analysis set population is equivalent to the safety analysis population.

The full analysis set population will be used mainly for all effectiveness and efficiency summaries and is considered the primary population. Descriptive statistics will be calculated using as reference the number of participants in the relevant analysis population, according to the nature of the data (i.e., continuous, discrete, time to event) as follows: continuous variables by number of non-missing observations, arithmetic mean, standard deviation, minimum and maximum values, median and quartiles and categorical variables by absolute and relative frequencies.

#### Study participants

Participant disposition including the number of participants enrolled/implanted and the numbers completing or discontinuing (including the reason for discontinuation) will be provided. Information on demographics, implant characteristics, medical history, medications, and treatments will be summarized as appropriate.

#### Primary analysis

The primary endpoint of response ─ defined as a decrease in MADRS total score of at least 50% from baseline to 12 months post-implant ─ will be provided for patients who were experiencing a major depressive episode at the time of enrolment (i.e. a baseline MADRS total score ≥ 20). The number and percentage of participants with response and the associated 95% confidence interval will be presented.

#### Secondary efficacy analyses

The secondary analyses will include response duration and changes in mood, severity of depression, mania, quality of life, psychosocial impairment, cognition, anxiety, suicidality, and adjunctive antidepressant treatment usage. Absolute scores and changes from baseline in each of the continuous secondary endpoints will be summarized for each of the follow-up assessments; and 95% confidence intervals for the changes will be presented.

#### Exploratory efficacy analyses

Further exploratory analyses of the primary endpoint may be conducted to identify and better understand any correlations between selected demographic, baseline characteristics, and response status using logistic regression techniques. Exploratory analyses of changes in positive affect, negative affect, hedonic tone, cognitive, and overall functioning, meaningfulness of life and happiness and patient functioning as measured by LAPS will be performed. Furthermore, exploratory analyses will be carried out with the aim to assess the resource consumption associated to VNS therapy for DTD management per patient; this will include hospitalizations, antidepressant medication usage and adjunctive antidepressant treatments pre and post VNS Therapy. For those patients who provide consent to collect additional data and who are enrolled in a country with a national healthcare database, a more detailed resources consumption analysis will be conducted.

#### Safety analysis

Safety data will be summarized descriptively, based on the safety analysis population (defined above). Adverse event data will be summarized by means of number of participants with at least one event, incidence rates, crude incidence rates. Similarly, statistics for serious adverse event; adverse device effect; serious adverse device effect; unanticipated serious adverse device effect and deaths will be presented.

Note that an adverse event with a ‘related’ causal relationship to the VNS therapy will be considered an Adverse Device Effect. Details of any recorded device deficiencies will be listed. Further safety tabulations may be presented, as appropriate.

#### Interim analysis

An interim analysis may be planned after a sufficient number of patients have enrolled into the study.

### Data monitoring

The principal investigators will oversee their respective centres, while the sponsor’s study manager will oversee both the scientific and administrative aspects of RESTORE-LIFE. The principal investigators will communicate regularly with the sponsor’s study manager regarding data collection, and the project coordinator will ensure smooth internal and external communications.

### Study steering committee

A steering committee has been established for this study to assist the sponsor in designing and managing the study based upon scientific, medical, and technical experience and expertise. The committee is comprised of individuals with specialized knowledge and experience in psychiatry and/or any other discipline needed to fulfil the responsibilities of the steering committee.

## Discussion

This paper describes the study protocol for RESTORE-LIFE: a prospective, observational, multi-site, global post-market study intended to assess short-, mid-, and long-term effectiveness and efficiency outcomes in ‘real-world’ setting among patients with DTD treated with VNS Therapy as adjunctive therapy.

To our knowledge, RESTORE-LIFE will be the largest post-market trial with VNS Therapy in DTD to be conducted globally.

The study has several strengths, it is multicentre and has been designed to evaluate if VNS Therapy meaningfully improves and sustains clinical and depressive symptom outcomes in patients with DTD. The study will also investigate the durability of VNS response in DTD and the long-term disease management of patients with DTD treated with VNS. In addition, the study results will potentially clarify clinical, functional, and health economics questions in a real-world patient population with DTD in different countries around the world. Changes in positive affect, negative affect, hedonic tone, cognitive functioning, overall functioning, meaningfulness of life, and happiness will also be assessed.

We anticipate that the study participants may benefit from more frequent visits with their treating physician. In addition, patients receiving VNS Therapy in the future may benefit from information gathered during this study outside the standard of care.

Currently there is no standard approach to the management of DTD as a standardized algorithm will not be appropriate for the treatment of such a heterogenous patient population. The results of this study may provide new evidence to influence clinical practice, including potentially providing access to VNS Therapy to patients with DTD in new and/or additional geographical regions, and help healthcare providers make more informed therapeutic decisions in the use of VNS Therapy as an effective and safe adjunctive therapy in treating patients with DTD.

As the study will assess VNS Therapy for the treatment of patients in a real-world setting, it may potentially have several related limitations, including the lack of randomization and blinding of participants and clinicians, the lack of a control group, potential attrition of participants and deviations in the delivery of the intervention, differential optimization of VNS parameter settings, differential use of concomitant anti-depressant medications and treatments in patients, and the heterogeneous study population may limit generalizability. The suicidality assessment may be limited as it will be assessed based on a single item in the MADRS scale and a single item in the QIDS-SR scale, instead of being assessed using a specific suicidality scale. In addition, this will be a 5-year longitudinal study and participant attrition over time may decrease the sample sizes at each analysis time point.

### Study status

Enrolment in the study commenced in December 2017 and is expected to take about 5 years to complete. At the time of submission, participant recruitment and data collection are ongoing with 76 study participants enrolled, and new centres are continuing to be selected for study participation. The RESTORE-LIFE study is running in parallel with a randomized blinded trial of VNS in TRD in the United States (RECOVER trial; ClinicalTrials.gov identifier NCT03887715) [50].

## Data Availability

No data is available at the time of submission as the study is in enrolment phase. After the dataset has been locked, the data will be available from the study sponsor LivaNova on reasonable request.

## Abbreviations

ASRM: Altman Self-Rating Mania Scale
ATHF: Antidepressant Treatment History Form
CE Marking: Conformité Européene (European Conformity)
DTD: Difficult-to-Treat Depression
ECT: Electroconvulsive Therapy
EQ-5D-5L: EuroQoL five dimensions questionnaire
FDA: Food and Drug Administration
GAD-7: Generalized Anxiety Disorder Assessment
LAPS: Leuven Affect and Pleasure Scale
MADRS: Montgomery Åsberg Depression Rating Scale
MINI: Mini-International Neuropsychiatric Interview
PDQ-5: Perceived Deficits Questionnaire
QIDS-SR: Quick Inventory of Depressive Symptomatology Self-Report
Q-LES-Q-SF: Quality of Life Enjoyment and Satisfaction Questionnaire – Short Form
rTMS: repetitive Transcranial Magnetic Stimulation
TAU: treatment-as-usual
THINC-it: THINC-Integrated Tool
TRD: Treatment-Resistant Depression
VNS: Vagus Nerve Stimulation
WPAI:D: Work Productivity and Activity Impairment Questionnaire-Depression
